# Red Is Not a Proxy Signal for Female Genitalia in Humans

**DOI:** 10.1371/journal.pone.0034669

**Published:** 2012-04-06

**Authors:** Sarah E. Johns, Lucy A. Hargrave, Nicholas E. Newton-Fisher

**Affiliations:** School of Anthropology and Conservation, University of Kent, Canterbury, Kent, United Kingdom; University of Jyväskylä, Finland

## Abstract

Red is a colour that induces physiological and psychological effects in humans, affecting competitive and sporting success, signalling and enhancing male social dominance. The colour is also associated with increased sexual attractiveness, such that women associated with red objects or contexts are regarded as more desirable. It has been proposed that human males have a biological predisposition towards the colour red such that it is ‘sexually salient’. This hypothesis argues that women use the colour red to announce impending ovulation and sexual proceptivity, with this functioning as a proxy signal for genital colour, and that men show increased attraction in consequence. In the first test of this hypothesis, we show that contrary to the hypothesis, heterosexual men did not prefer redder female genitalia and, by extension, that red is not a proxy signal for genital colour. We found a relative preference for pinker genital images with redder genitalia rated significantly less sexually attractive. This effect was independent of raters' prior sexual experience and variation in female genital morphology. Our results refute the hypothesis that men's attraction to red is linked to an implied relationship to genital colour and women's signalling of fertility and sexual proceptivity.

## Introduction

Red is a colour that induces both physiological and psychological effects in humans, affecting sporting and other competitive success, signalling or enhancing male social dominance [1]–[5]. There is also a broad view, based primarily on work by Masters and Johnson [6], that red, particularly red sexual skin, is indicative of sexual willingness and a defining characteristic of human female sexual arousal [6]–[11].

Artificial stimuli can exploit instinctive behavioural responses [12], and the link between the colour red and sexual attraction appears to be widely cross-cultural, with brothels and sex workers utilising this colour as a symbol of their trade, and lingerie, lipstick, and Valentine's Day cards predominantly featuring red as the colour of choice [10], [13], [14]. Men appear to regard a woman as more sexually attractive and desirable if she is associated with red objects or contexts [14]–[17] and it has been proposed that human males have a biological predisposition towards the colour red such that it is ‘sexually salient’ [8], [9], [14], [15], [18]. It has been suggested that women seek to remind men of their red, aroused labia by displaying or wearing the colour red, especially in their use of red lipstick [9], [18]–[21]. Evidence for the importance of red in human mating behaviour and sexual interactions has come from studies of the impact of background colours [14], [17] and clothing colour [14]–[16], or discussions of female cosmetic use [9], [18]–[21], on judgements of attractiveness, rather than images of the sexual skin for which these are assumed to be a proxy.

Humans share close phylogenetic relationships with other primate species that show enlarged, conspicuous sexual skins to signal sexual receptiveness. These swollen sexual skins occur in a variety of old-world primates; their function is not clearly understood, although a variety of hypotheses have been explored [22]. Recently there have been suggestions that the size and the shape of the swellings are honest signals of female condition and genetic quality [23], [24]. For example, male chacma baboons (*Papio ursinus*) prefer females whose ornaments are larger. There is also evidence that some primate males have a preference for red as the colour of these swellings. Chacma baboons show increased levels of masturbation when presented with ovariectomised females fitted with red, artificial sexual swellings [25] and male rhesus macaques (*Macaca mulatta*) spend significantly longer looking at red-enhanced images of the female anogenital region and surrounding skin [26]. Although the function(s) of brightly coloured, conspicuous sexual skins may differ in different primate clades [22] and conspicuousness of the swellings varies between individuals within a species [27], the ‘sexually salient’ hypothesis argues that, as with females in many old world primate species, women use the colour red to announce impending ovulation and sexual proceptivity, with red stimuli functioning as a proxy signal for sexually receptive genital colour. In consequence men find ‘red’ attractive and favour this colour during sexual interactions [8], [14], [15], [18]. There are, however, no studies in humans that parallel the tests of genital colour preference in non-human primates.

There are few systematic data concerning the characteristics of normal human female genitalia, including colour and, perhaps surprisingly given its persistence in popular science accounts, the ‘sexually salient’ hypothesis remains untested. The available evidence for this hypothesis, as summarised above, is either circumstantial or indirect, or based on simple cross-species comparisons. Only four studies have attempted to quantify variation in normal, external adult female genital morphology [28]–[31], and only two studies, since Masters and Johnson [6], have specifically studied variation in female genital colour. Analysis of Playboy Magazine centrefolds from 1957–2007 did not find any change in labia minora colour (described as pink or light red) over time, even though other features, such as amount of pubic hair, varied [32], while Lloyd et al. [29] compared labial colour to surrounding skin (whether it was lighter or darker). While there is evidence for an impact of red on judgements of sexual attractiveness, it is not clear that such evidence provides support for the ‘sexually salient’ hypothesis, or how it can be reconciled with the strong cross-species evidence [1], [12], [33], [34] for red as a signal of social dominance.

To resolve at least part of this quandary, we present a direct test of the central assumption of the ‘sexually salient’ hypothesis, that artificial red signals are a proxy for female sexual skin, by investigating whether men's preference for red applies to the sexual skin in the vulva region. If men find women displaying red more attractive because the coloured stimuli ‘hijack’ a biological response towards female genitalia, such that men respond to it as a signal of fertility or arousal, we should see a preference for red when viewing female genitalia directly. If the ‘sexually salient’ hypothesis holds, males should either prefer redder sexual skin to other shades, or show a step increase in their ratings of attractiveness with increasing redness.

## Materials and Methods

We generated 16 images of female genitalia by manipulating four individual photographs of the human female vulva, such that we had four colour conditions for four different base images. We used colours within the normal range expected for human genitalia, a gradation of increasing redness starting from a pale pink. This was done to prevent any aversion in our participants (described below) to ‘unnatural’ images [26]: for instance, some NHP studies of genital colour preference have contrasted red with anatomically atypical block colours (e.g. green, purple, orange) [25].

Explicit images of anatomically normal, un-retouched, non-pornographic, similarly-orientated female genitals were surprisingly difficult to obtain, and the number of images used in the experiment reflects this difficulty. We obtained the photographs from a female genital image comparison website (www.vulvavelvet.org), the purpose of which is to inform and educate the public about natural variation in human female genitalia, and to make women feel comfortable with their bodies. Women, over the age of 18, anonymously donate images of their genitals to this website, placing them in the public domain. We selected photographs that were taken from similar angles, did not contain other, potentially distracting, objects (fingers, sex toys, piercings etc.) and were hairless to account for current fashion [32]. The ratio of the length of the labium majus to the length of the labium minus [30], [31] in each of the four selected photos was broadly comparable (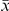 = 1.27+/−0.13), but there was, naturally, some individual variation in the overall morphology and protuberance of the labia minora. We controlled for this morphological variation in subsequent analysis by including each of the four images explicitly as a within-subject factor (vulva morphology) in the model. Permission to use the images was obtained from the owner/editor of the website, with the caveat that complete, un-manipulated images would not be presented in publication. The fact that the website had a named individual who we could contact to request permission to use the images was also an important factor in our image selection process.

**Figure 1:**
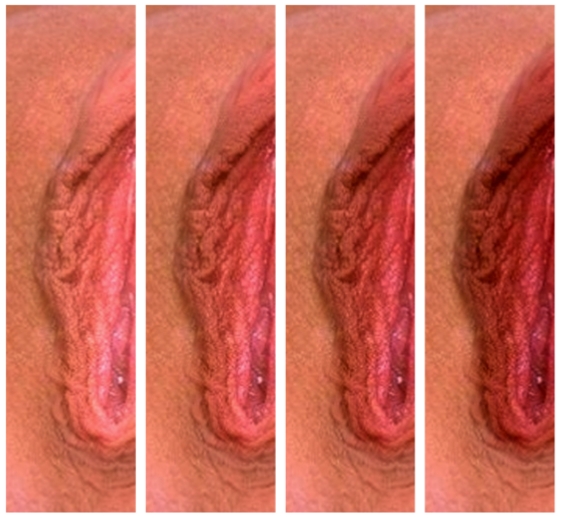
Right labium minus of vulva base image no. 2. The 4 different colour conditions used in the experiment are shown. To account for unpredictable colour variation between computer monitors and printers, colour conditions were calibrated to the computer monitors used in this study. Left to right: Pale pink, Light pink, Dark pink, Red.

We used Microsoft PhotoDraw v2 to crop out the labia minora and clitoris from the four photographs, and re-coloured these through a trial and error process of manipulating the colour balance and the brightness [14]. In order to account for unpredictable colour variation between computer monitors and printers, re-colouring was conducted to present appropriately on the monitors on which the images would be displayed to participants. The manipulated section of each vulval image differed only in brightness, presenting a subtle gradation of four different colour conditions - pale pink, light pink, dark pink, and red (Figure 1). This allowed us to preserve variation in contrast and morphological details found in the original images. The cropped area was then pasted on top of the original photograph, with ‘softened edges’ to disguise the manipulation. It is worth restating that we were not interested here in exploring male response to the natural variation in vulval morphology or colour, but in testing the male response to red within the context of female genitalia. Thus all images were re-coloured, and we did not present subjects with un-manipulated images, which also allowed us to avoid an influence of manipulation *per se* on participant response.

We presented these sixteen manipulated images to forty males (Age: 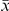 = 21.13+/−5.46 years) without any colour vision deficiencies. Images were shown to participants once, and in a semi-randomized order such that each sequential image showed a different vulva and colour condition, as an untimed Microsoft PowerPoint slideshow. Each participant rated the sexual attractiveness of each image using a 100 mm visual analogue scale (0 = Unattractive, 100 = Attractive). This VAS rating method was selected because it provides a continuous, unconstrained scale in contrast to the more commonly employed Likert scale [35]. We tested for order effects, in case participants became desensitised or habituated to the images. The participants also provided information on sexual orientation, again using a visual analogue scale, their age, and number of previous sexual partners. This study and its data collection protocol were approved by the Research Ethics Advisory Group of the School of Anthropology and Conservation, University of Kent. The participants were informed that they could withdraw from the study at any point and were warned of the graphic nature of the vulva images before participating. All data was collected and analysed anonymously, and written consent was obtained from all participants.

We analysed these data using a repeated measures general linear model (Mixed between-within subject ANOVA). Genital colour (*pale pink*, *light pink*, *dark pink*, *red*) and vulva morphology (*base image 1* through *4*) were within-subjects factors; participants' sexual experience (*very little* (n = 20, 0–2 sexual partners) or *some* (n = 20, 3+ partners)) was a between-subject factor. While there was considerable skew in the number of reported previous sexual partners (skew/s.e. skew = 4.02, Shapiro-Wilk *W* = 0.813, p<0.001), we decided that two equal sized groups (representing 1: *very little* or 2: *some*) best captured participants' experience. Given this somewhat arbitrary decision, we also explored other categorisations of this variable, but none of these led to different results. We conducted analysis using PASW (SPSS) 18. Data were suitable for parametric analysis, we set α to 0.05 and used Bonferroni adjustments for multiple comparisons. Means are presented with standard deviations.

## Results

All males who participated in the study reported being heterosexual (VAS score: 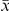 = 92.28+/−10.62). We found that men presented with images digitally manipulated to show a gradation in colour from light pink to red (Figure 1), rated the reddest shade least attractive. Colour exerted a significant effect on the attractiveness of the images (Wilks' Lambda = 0.59, F_3,36_ = 8.25, p<0.001, partial η^2^ = 0.40) with red rated (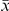 = 35.37+/−21.61) significantly less attractive than the three pink shades (Bonferroni pairwise comparisons: pale: p = 0.001; light: p = 0.001; dark: p = 0.002), among which there was no significant difference in rated attractiveness (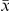 = 40.32+/−23.21). We found no order effects: there was no significant relationship between the mean score an image received and the position in which it was shown to participants in the slideshow (*r*_s_ = −0.109, n = 16, p = 0.68). Later presented images did not receive higher scores due to participant desensitisation. Our analysis controlled for the variation in vulva morphology on attractiveness ratings: there was no significant interaction between genital colour and vulva morphology (Wilks Lambda = 0.62, F_3, 36_ = 1.98, p = 0.08). We also controlled for participants' previous sexual experience (number of previous sexual partners 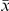 = 3.92+/−4.28; range 0–17), which did not significantly influence the attractiveness ratings (F_1,28_ = 0.48, p = 0.49, partial η^2^ = 0.01).

## Discussion

Our results challenge the ‘sexually salient’ hypothesis, and the general view that red promotes sexual attractiveness by acting as a proxy for genital colour. Neither of the predictions that we derived from the sexually salient hypothesis were supported: men showed neither a preference for the reddest shade, in fact finding this least attractive, nor a step increase in their attractiveness ratings with increasing redness. Our results lead us to seriously question the persistence of this hypothesis.

We found a strong aversion to red as the colour of female genitals. These are not an easily displayable ‘badge’, given our bipedal posture. It is unlikely that human females use their genital skin as a signal to recruit mates, as is the case for baboons and macaques, so their colour can do nothing to aid male social dominance or female competition. Instead, colour preference may relate to detection of disease risk, and/or mate value. Pinker vulval skin is associated with youth (after menopause it becomes paler [36]) and not being pregnant (when blood volume heightens colour [37]). Surprisingly little is known about the range of variation in morphology and colour of the external genitalia of normal women of reproductive age, however, and further research is necessary in this area.

Aversion to images of redder vulvas may stem from an association between the colour red and menstrual blood [38]. Disgust and phobia toward blood in general is a common human response [39], [40], as are strong cross-cultural taboos surrounding menstruation [41], [42]. Signs of menstruation could also indicate lowered female sexual arousal and willingness [43]. Menstruation is also a period of virtual infertility, and is a clear signal that male mating effort is highly unlikely to be successful. Mating males may also suffer increased disease risk from menstrual blood exposure [44]. There are also multiple health conditions that cause bright red vulval skin across a menstrual cycle [45], including vulvovaginal candidosis [46] and trichomoniasis [47] although it is worth noting that our reddest shade did not approach the intensity and brightness of the red colouration associated with either of these conditions or with fresh menstrual blood.

If men show an aversion to red in the context of female genitalia because it signals increased disease risk to themselves, poor female condition, or sub-fertility, it is difficult to defend a hypothesis that holds that the increased attractiveness of women associated with strong red signals is a response to a proxy signal for genitalia. This ‘sexually salient’ hypothesis is also difficult to reconcile with studies that suggest red functions as a signal of male quality [1], that artificial red badges enhance male dominance [1]–[3], and that viewing red is associated with failure and lack of success [4], [5].

Nevertheless, there are sound data supporting an association between red and judgements of sexual attractiveness. One possibility is that the effect of red on male judgements of sexual attractiveness is related to male efforts to enhance social dominance. Men may work harder to acquire objects that they can display as red badges of dominance, and so be motivated to compete more strongly for women associated with red. Women may even use red (clothing, cosmetics) to stimulate such competition as a means of selecting higher quality mates [14]. Our results should encourage future studies, into the relationship between the colour red and sexual attractiveness, to move on from ideas such as the “sexually salient” hypothesis.

Colours are important signals in many non-human species. They influence mood and emotion in humans, and red, in particular, has important effects on male behaviour. Our study shows that ideas of red as a proxy for female genital colour, and naïve cross-species comparisons, must be replaced by careful consideration of precisely what is being signalled and how those signals are interpreted. Our findings have important ramifications for the future study of the role of colour signals in human social and sexual interactions.

## References

[1] Hill RA, Barton RA (2005). Red enhances human performance in contests.. Nature.

[2] Attrill M, Gresty K, Hill R, Barton R (2008). Red shirt colour is associated with long-term team success in English football.. J Sports Sci.

[3] Little A, Hill R (2007). Attribution to red suggests special role in dominance signalling.. J Evol Psychol.

[4] Ilie A, Ioan S, Zagrean L, Moldovan M (2008). Better to be red than blue in virtual competition.. Cyberpsychol Behav.

[5] Maier MA, Elliot AJ, Lichtenfeld S (2008). Mediation of the negative effect of red on intellectual performance.. Pers Soc Psychol B.

[6] Masters WH, Johnson VE (1966). Human sexual response.

[7] Kinsey A, Pomeroy W, Martin C, Gebhard P (1953). Sexual behavior in the human female.

[8] Morris D (1967). The naked ape.

[9] Morris D (2005). The naked woman.

[10] Pallingston J (1999). Lipstick: a celebration of a girl's best friend.

[11] Heffner LJ, Schust DJ (2010). The reproductive system at a glance.

[12] Cuthill IC, Hunt S, Cleary C, Clark C (1997). Colour bands, dominance, and body mass regulation in male zebra finches (*Taeniopygia guttata*).. Proc R Soc B.

[13] Greenfield AB (2006). A perfect red: empire, espionage, and the quest for the color of desire.

[14] Elliot AJ, Niesta D (2008). Romantic red: red enhances men's attraction to women.. J Pers Soc Psychol.

[15] Niesta Kayser D, Elliot AJ, Feltman R (2010). Red and romantic behavior in men viewing women.. Eur J Soc Psychol.

[16] Roberts SC, Owen RC, Havlicek J (2010). Distinguishing between perceiver and wearer effects in clothing color-associated attributions.. Evol Psychol.

[17] Jung I, Kim MS, Han K (2011). Red for romance, blue for memory: HCI International 2011 – posters' extended abstracts.. Comm Com Inf Sc.

[18] Low BS, Chagnon N, Irons W (1979). Sexual selection and human ornamentation.. Evolutionary biology and human social behaviour.

[19] Ackerman D (1993). A natural history of the senses.

[20] Brewer S, Webb T (2004). The bluffer's guide to sex.

[21] Stephen ID, Coetzee V, Law Smith M, Perrett DI (2009). Skin blood perfusion and oxygenation colour affect perceived human health.. PLoS ONE.

[22] Nunn CL (1999). The evolution of exaggerated sexual swellings in primates and the graded-signal hypothesis.. Animal Behaviour.

[23] Huchard E, Courtiol A, Benavides JA, Knapp LA, Raymond M (2009). Can fertility signals lead to quality signals? Insights from the evolution of primate sexual swellings.. Proc R Soc B – Biol Sci.

[24] Huchard E, Raymond M, Benavides J, Marshall H, Knapp L (2010). A female signal reflects MHC genotype in a social primate.. BMC Evol Biol.

[25] Bielert C, Girolami L, Jowell S (1989). An experimental examination of the colour component in visually mediated sexual arousal of the male chacma baboon (Papio ursinus).. J Zool.

[26] Waitt C, Gerald MS, Little AC, Kraiselburd E (2006). Selective attention toward female secondary sexual color in male rhesus macaques.. Am J Primatol.

[27] Anderson CM, Bielert CF (1994). Adolescent exaggeration in female catarrhine primates.. Primates.

[28] Verkauf BS, Thron JV, O'Brien WF (1992). Clitoral size in normal women.. Obstet & Gynecol.

[29] Lloyd J, Crouch NS, Minto CL, Liao L-M, Creighton SM (2005). Female genital appearance: ‘normality’ unfolds.. BJOG – Int J Obstet Gy.

[30] Basaran M, Kosif R, Bayar U, Civelek B (2008). Characteristics of external genitalia in pre- and postmenopausal women.. Climacteric.

[31] Howarth H, Sommer V, Jordan FM (2010). Visual depictions of female genitalia differ depending on source.. Med Humanities.

[32] Schick VR, Rima BN, Calabrese SK (2011). Evulvalution: the portrayal of women's external genitalia and physique across time and the current Barbie doll ideals.. J Sex Res.

[33] Setchell JM, Jean Wickings E (2005). Dominance, status signals and coloration in male mandrills (Mandrillus sphinx).. Ethology.

[34] Pryke S, Griffith S (2006). Red dominates black: agonistic signalling among head morphs in the colour polymorphic Gouldian finch.. Proc R Soc B – Biol Sci.

[35] Crichton N (2001). Visual analogue scale (VAS).. J Clin Nurs.

[36] Stika CS (2010). Atrophic vaginitis.. Dermatol Ther.

[37] Farage M, Maibach H (2006). Lifetime changes in the vulva and vagina.. Arch Gynecol Obstet.

[38] Knight C (1995). Blood relations: menstruation and the origins of culture.

[39] Schienle A, Stark R, Walter B, Vaitl D (2003). The connection between disgust sensitivity and blood-related fears, faintness symptoms, and obsessive-compulsiveness in a non-clinical sample.. Anxiety Stress Copin.

[40] Ayala ES, Meuret AE, Ritz T (2009). Treatments for blood-injury-injection phobia: a critical review of current evidence.. J Psychiat Res.

[41] Buckley T, Gottlieb A (1988). Blood magic: the anthropology of menstruation.

[42] Kissling EA (2002). On the rag on screen: menarche in film and television.. Sex Roles.

[43] Gangestad SW, Thornhill R, Garver-Apgar CE (2010). Fertility in the cycle predicts women's interest in sexual opportunism.. Evol Hum Behav.

[44] Tanfer K, Aral SO (1996). Sexual intercourse during menstruation and self-reported sexually transmitted disease history among women.. Sex Transm Dis.

[45] Edwards L (2004). The diagnosis and treatment of infectious vaginitis.. Dermatol Ther.

[46] Morton RS, Rashid S (1977). Candidal vaginitis: natural history, predisposing factors and prevention.. P Roy Soc Med.

[47] Wølner-Hanssen P, Krieger JN, Stevens CE, Kiviat NB, Koutsky L (1989). Clinical manifestations of vaginal trichomoniasis.. JAMA – J Am Med Assoc.

